# Production and characterization of anti-bacterial metabolite(s) from Egyptian archaeological sites

**DOI:** 10.1038/s41598-025-06670-x

**Published:** 2025-06-27

**Authors:** Aalaa A. Youssef, Bassma H. Elwakil, Doaa A. Ghareeb, Zakia A. Olama

**Affiliations:** 1 Botany and Microbiology Department, Faculty of Science, Alexandria University, Alexandria, 21500 Egypt; 2 Medical Laboratory Technology Department, Faculty of Applied Health Sciences Technology, Pharos University in Alexandria, Alexandria, Egypt; 3 Bio-Screening and Preclinical Trial Lab, Biochemistry Department, Faculty of Science, Alexandria University, Alexandria, Egypt; 4 Center of Excellence for Drug Preclinical Studies (CE-DPS), Pharmaceutical and Fermentation Industry Development Center, City of Scientific Research and Technological Applications (SRTA-City), New Burg El-Arab City, Alexandria Egypt; 5 Research Projects Unit, Pharos University in Alexandria, Alexandria, Egypt

**Keywords:** *Xenorhabdus nematophila*, Bioactive producer, Nano-xenortide, Optimization, Antibacterial, Anticancer, Cancer, Infectious diseases, Nanobiotechnology, Nanoscale materials

## Abstract

Antimicrobial agents produced by *Xenorhabdus* spp. may hold the answer to novel antimicrobial agents. Antibacterial activity of some bacterial strains isolated from different Egyptian archaeological sites was evaluated. The most potent organism that reported high antibacterial activity was identified as *Xenorhabdus nematophila*. The produced bioactive compound was identified as xenortide using LC–MS and NMR studies. Optimization of xenortide’s production was assessed using a central composite statistical design. The most effective fermentation factors were identified as carbon, nitrogen source concentrations and pH levels. Nano-xenortide was synthesized using the ball milling method, followed by its characterization and evaluation for its anticipated antibacterial and anticancer properties. Statistical analysis of the findings indicated that the produced nano-xenortide exhibited superior antibacterial efficacy. Furthermore, the assessment of its cytotoxicity revealed that nano-xenortide is a promising, safe candidate that can be used as an antibacterial and anti-colorectal-carcinoma agent.

## Introduction

Multidrug-resistant (MDR) bacteria pose a severe hazard to the entire world. Most of the MDRs show complete resistance to antibiotics, resulting in significant limitations in treatment options for many cases. Hence, there is an urgent need for more effective antimicrobial agents to combat MDR infections, although traditional antimicrobial drugs are relatively effective against some communicable diseases^1^. Antibiotics have become less and less effective, with high-risk side effects. Moreover, multidrug-resistant microbes such as Methicillin-resistant *Staphylococcus aureus* (MRSA), *Escherichia coli, Klebsiella pneumoniae,* and *Pseudomonas aeruginosa* find new mechanisms of defence and resistance against the emerging antibiotics. Therefore, a worldwide intensive search for new antibiotics is urgently needed^2^.

*Xenorhabdus* spp. produces various bioactive compounds throughout its growth cycle, and the genus is an underestimated and neglected source of novel bioactive compounds with various chemical structures^3^. Biologically active compounds produced by *Xenorhabdus* spp. exhibit a wide range of activities, including the inhibition of bacterial growth, fungi and protozoa, as well as the suppression of insect and nematode development and the prevention of cancer cell formation.^4^. These natural metabolites provide valuable clues in the research and development of drugs and agrochemicals. The secondary metabolites of *Xenorhadbus* spp. contain a wide range of antimicrobial products namely depsipeptides such as xenematides, xenomacins, szentiamide and xenobactin, lipodepsipeptides, xenocoumacins, fabclavines, pristinamycin, xenortides, rhabdopeptides, bicornitun, PAX peptides, cabanillasin, nemaucin, dithiolopyrrolone derivatives, indole-containing compounds, benzylideneacetone, rhabdicin, bacteriocins and a few unnamed peptides that are still under investigation^3^.

On the other hand, nanotechnology is currently being utilized to explore various avenues of medical science in several ways. New-era drugs are nanoparticles of polymers, metals, or ceramics that can combat medical dilemmas, such as cancer, as well as fight human pathogens^5^. In addition to the importance of the nanocarriers in drug delivery and their physicochemical properties, they also enhance drug performance by improving solubility, degradation, clearance, targeting, theranostics, and combination therapy^6^. Ball milling is a mechanical process that facilitates intentional physical and chemical transformations in powdered materials. The method is based on evidence that shows the physical and chemical behaviours of individual molecules and ordered and disordered solids can be affected by non-hydrostatic mechanical stresses and the associated strains^7^. One of the most essential advantages of the ball milling technique is that it is performed at room temperature, thereby avoiding the limitations associated with high temperatures and the use of toxic solvents.

Additionally, ball milling provides an efficient and cost-effective method for industrial manufacturers to synthesize nanoparticles, particularly for temperature-sensitive molecules^8^. The present study aimed to focus on isolating antimicrobial-producing bacteria from Egyptian archaeological sites and identifying their biologically active compound(s). Then, novel nanoparticles will be synthesized from the identified biologically active compounds.

## Materials and methods

### Microorganisms

Different bacterial and fungal multidrug-resistant (MDR) pathogens were used throughout the present work, namely: Methicillin-resistant *Staphylococcus aureus* (MRSA), *Staphylococcus aureus, Escherichia coli, Candida albicans, Klebsiella pneumoniae, Pseudomonas aeruginosa, Proteus vulgaris, and Acinetobacter baumannii.* All strains were provided by the Surveillance Microbiology Department of Pediatric Al-Shatby Hospital in Alexandria and were identified using the VITEK system (BIOMERIEUX, USA). *Xenorhabdus nematophila* was isolated from Egyptian archaeological sites and was identified by 16S rRNA.

### Antimicrobial agents producing bacteria

#### Isolation

Soil Samples were collected from different sites using standard methods at Luxor, Aswan, Edfu, Komombo, and Philea temples. Each sample was sieved, and 1 g of soil was added to sterilized test tubes containing 9 mL sterile physiological saline (NaCl, 8.5 g/L), with shaking using a vortex mixer. The soil suspensions were serially diluted and cultivated and incubated at 35 ± 2 °C for 72 h on nutrient agar (NA) (Merck Co., NJ, USA). Different bacterial colonies were selected and maintained as pure cultures on NA slants with periodic transfer to fresh media.

This research was granted for sample collection and publishing by the Institutional Animal Care and Use Committees (IACUCs) at the Faculty of Science, Alexandria University, with approval number: AU04-622-30-05-2023.

#### Screening for antimicrobial agents producing bacteria

The isolated bacteria were grown in nutrient broth under shaken conditions (150 rpm/min) for 48 h at 35 ± 2 °C. After incubation, the bacterial suspension was centrifuged at 5000 × g for 15 min. The supernatant was collected carefully and filtered through a 0.22 μm Millipore filter (ISOLAB). An aliquot (50 µL) of the filtrate was then streaked onto NA agar to verify the absence of bacterial cells. Bacterial supernatants were assessed for their antimicrobial effect against different multidrug-resistant (MDR) pathogens, namely Methicillin-resistant *Staphylococcus aureus* (MRSA), *Staphylococcus aureus, Escherichia coli, Candida albicans, Klebsiella pneumoniae, Pseudomonas aeruginosa, Proteus vulgaris,* and *Acinetobacter baumannii,* using the disc-diffusion method according to Afifi and Khabour^9^. The agar plates were prepared and inoculated with the tested bacteria at a 5 × 10^6^ CFU/mL concentration. Each sterilized disc (Whatman No. 1 with 6 mm diameter) was saturated with 25 μl of 20 mg filtered suspension under test, then placed over the inoculated plates. Isolated organisms with the highest potential for antimicrobial activity were selected for further investigations.

#### Identification of the most promising antimicrobial agents producing bacteria

The most promising bacterial isolate exhibiting the highest antimicrobial activity was identified phenotypically and genotypically. After sequencing of the 16S rRNA, a multiple sequence alignment was performed in accordance with the National Center for Biotechnology Information (NCBI) database. Finally, the phylogenetic tree for the promising isolate was generated through distance matrix analysis using the NT system.

### Optimization of the antimicrobial activity

#### Optimization using one factor at a time

In a trial to select the most promising carbon and nitrogen sources that maximize the production of the antimicrobial agent, different carbon sources (0.5%) (sucrose, lactose, Glucose, Starch, and Mannitol) and nitrogen sources (0.3%) (peptone, tryptone, casein, yeast, and Urea) were evaluated individually. Bacterial supernatant was collected after 48 h of incubation under shaken conditions and tested for antimicrobial activity using the disc diffusion method. The best-optimized carbon and nitrogen sources were selected for further experiments.

#### Optimization using central composite design (response surface methodology)

A central composite design (CCD) was applied to study and optimize the most influential variables that affected the antimicrobial activity. Moreover, interactions between multiple parameters were evaluated by employing statistical modelling with response surface methodology (RSM), which uses a statistical approach with five levels (− 2.37841, − 1, 0, 1, and 2.37841) to assess the effect of the conditions’ variations in different parameters, to detect the optimal conditions (Table 1). The environmental and nutritional factors of the most promising isolate, namely the carbon source level, nitrogen source level, pH, temperature, and incubation time, were optimized to maximize the antimicrobial activity of the bacterial supernatant (R1). The mathematical relationship of the response of these parameters can be illustrated by a quadratic (second degree) polynomial equation (Eq. 1), where y is the response value; b0 is the constant; x1, x2, x3, x4, and x5 are the independent parameters; b1, b2, b3, b4, and b5 are the linear coefficients; b12, b13, b14, b15, b23, b24, b25, b34, b35, and b45 are the cross-product coefficients; and b11, b22, b33, b44, and b55 are the quadratic coefficients. A total of fifty runs were processed to estimate the coefficients of the model using multiple linear regressions. The design of the experiments was conducted using Design-Expert 12.0®.

1 $$\begin{aligned} {\text{y }} = & {\text{ b}}0 \, + {\text{ b1x1}} + {\text{ b2x2 }} + {\text{ b3x3 }} + {\text{ b4x4 }} + {\text{ b5x5 }} + {\text{ b11x 2 1 }} \\ \quad & + {\text{ b22x 2 2 }} + {\text{ b33x 2 3 }} + {\text{ b44x 2 4 }} + {\text{ b55x 2 5 }} + {\text{ b12x1x2 }} + {\text{ b13x1x3}} \\ \quad & + {\text{ b14x1x4 }} + {\text{ b15x1x5 }} + {\text{ b23x2x3 }} + {\text{ b24x2x4 }} + {\text{ b25x2x5 }} + {\text{ b34x3x4 }} + {\text{ b35x3x5 }} + {\text{ b45x4x5}} \\ \end{aligned}$$

**Table 1:** Coded levels of the central composite analysis.

Parameters	Coded levels				
	− 2	− 1	0	1	2
Carbon source (%) (sucrose)	0.1	0.3	0.5	0.7	0.9
Nitrogen source (%) (yeast)	0.1	0.2	0.3	0.4	0.5
Incubation time (Days)	1.0	2.0	3.0	4.0	5.0
PH	5.0	6.0	7.0	8.0	9.0
Temperature (°C)	15.0	20.0	25.0	30.0	35.0

### Characterization of bioactive compounds using liquid chromatography–mass spectrometry (LC–MS), and nuclear magnetic resonance (NMR) analyses

The most potent antimicrobial agent characterization was conducted using liquid chromatography-mass spectrometry (LC–MS), and NMR after separation with column chromatography and testing the eluent(s) activity. The NMR spectra were obtained using Bruker spectrometers (500 and 400 MHz). On the other hand, LC–MS analysis (LCMS-8060NX, Shimadzu Co., Kyoto, Japan) and the ESI–MS positive and negative ion acquisition mode was carried out on a XEVOTQD triple quadruple instrument (Waters Corporation, Milford, MA01757 U.S.A), which were conducted using a C-18 octadecylsilane column (1.7 µm − 2.1 × 50 mm) with a dual UV detector and binary pump. The elution solvents were (a) water/0.1% formic acid and (b) acetonitrile/0.1% formic acid. The fractions eluted with a flow rate of 0.2 mL\min and were further subjected to mass spectrometry (MS) analysis. MS analysis of the samples was conducted over a mass range of 50–1500 m/z.

### Nano-synthesis using the ball milling technique

The optimized bioactive product was introduced to the ball mill for nanosized fabrication. Physical synthesis experiments were performed with a FRITSCH planetary ball mill (Pulverisette 7 premium line, two hardened steel vials, 80 cm^3^ volume, and charged with 60 hardened steel balls that were 3 mm in nominal diameter as the milling bodies). Two grams of the lyophilized bioactive product were used as the precursor material. The angular velocity of the milling was fixed at 200 rpm for 30 min^10^.

#### Physicochemical characterization of the synthesized nanoparticles

The dynamic light scattering (DLS) technique was used to evaluate the Zeta potential, particle size (PS), and polydispersity index (PDI) of the synthesized nanoparticles using a Malvern Zetasizer according to Elnaggar et al.^11^. The FTIR spectrum of the synthesized nanoparticles was also analyzed using Perkin–Elmer R79521 FTIR (USA). The shape, size, and ultra-structure of the synthesized nanoparticles were examined using a transmission electron microscope (TEM) (JEM-100 CX, JOEL, Tokyo, Japan) (resolution 3 nm at 30 kV).

#### Antimicrobial activity of the synthesized nanoparticles

The synthesized nanoparticles were evaluated for antimicrobial activity against different pathogens using the disc-diffusion method and minimum inhibitory concentration (MIC). The effect of the synthesized nanoparticles on different antibiotics was also tested. The bacterial lethality curve was also assessed, and a confocal microscopic study of the microbial-treated cells was conducted.

### Cytotoxic effect

Different concentrations of the potent bioactive compound and its synthesized nanoparticles were tested for their effects on various cell lines. In a 96-well tissue culture plate, Vero, WI38, and Caco-2 cells (cell lines purchased from ATCC, CCL-81, CCL-75, and HTB-37, respectively) were plated each in their respective culture media at a density of 5000 cells/well. The tested materials were serially diluted and added to the cell monolayers, which covered the entire surface of 96-well microtiter plates. The microtiter plates were placed in a humidified incubator with 5% CO_2_ and kept at 37 °C for 24 h. The control cells were incubated without the tested materials. After incubating the cells, the viable cell yield was determined using a colorimetric technique. The quantification of live cells was performed using the MTT assay after a 24-h incubation period. In summary, the media in the 96-well plate was replaced with 100 µl of new culture RPMI 1640 medium without phenol red. Next, 5 mg of MTT in 1 mL of PBS was added to each well, including the untreated controls. The 96-well plates were placed in an incubator set at 37 °C with a carbon dioxide concentration of 5% for 4 h. An 85 µl portion of the medium was taken out of the wells, and 50 µl of DMSO was added to each well and mixed completely using the pipette. The mixture was then kept at 37 °C for 10 min. The colour intensity was measured at 490 nm in a microtiter plate reader spectrophotometer. Using the relation between the used concentrations and the neutral red intensity value, the IC_50_ of each compound was calculated^12^. Moreover, the selectivity index was calculated as follows:

$${\text{Selectivity}}\,{\text{ index }} = {\text{ IC5}}0 \, \,{\text{of}}\,{\text{ normal }}\,{\text{cells}}/{\text{IC5}}0\,{\text{ of}}\,{\text{ cancer }}\,{\text{cells}}$$

### Statistical analyses

The Design-Expert 12.0® software package from Stat-Ease was used to implement the design of the experiments using multifactorial Central Composite Design (CCD). Fisher’s Least Significant Difference (LSD) post hoc test was applied after running Analysis of Variance (ANOVA) to analyze and identify the pairs of means that were statistically different according to the t-values. The results were the means of three trials. The means of the treatments were considered significant when *p* < 0.05.

## Results and discussions

### Isolation of bacterial strains

Twelve different bacterial strains were isolated from different soil samples collected from Luxor, Aswan, Edfu, Komombo and Philea temples (Table 2). A total of four different isolates were isolated from the Aswan temple. The isolates from Komombo temple were motile with variable biochemical responses. Rizk et al.^13^ studied the microbial ecology of the Memphis necropolis’s Djoser and Lahun Pyramids using amplicon-based metabarcoding and culture-dependent isolation. It was found that *Blastococcus aggregatus, Blastococcus saxobsidens,* and *Blastococcus sp.* were the most prevalent stone-inhabiting bacteria. *Knufia karalitana*, *Pseudotaeniolina globosa*, and an unknown Sporormiaceae species were the dominant rock-inhabiting fungi. These microbes can cause physical and chemical damage and need microbiological conservation programs. In another study, microbial isolates were isolated from three locations in Cairo, Egypt, namely Mohamed Ali palace, El-Ghory Mosque and Mosque of El-Kady Abdel-Basetit. It was found that genus *Aspergillus* spp. was the most predominant organism isolated from all swabs of deteriorated marble, followed by *Acremonium* and *Penicillium* spp. Moreover, *Streptococcus thermophilus*, Bacillus *brevis*, *B. coagulans*, and one actinomycete *Nocardia* *asteroide* were also isolated^14^. Abd-Elhalim et al.^15^ identified three fungal isolates and one bacterial isolate were obtained from stone monuments in Temple of Hathor, Luxor, Egypt and were identified as *A. niger* isolate Hathor 2, *C. fioriniae* strain Hathor 3, *P. chrysogenum* strain HATHOR 1, and *L. sphaericus* strain Hathor 4, respectively.

**Table 2:** Morphological and biochemical characteristics of the bacterial isolates.

Isolate no	Source	Colony morphology	Gram Stain	Cell shape	Motility	Biochemical parameters					
						Catalase activity	Coagulase activity	Oxidase	Gelatin liquefaction	Indole production	Nitrate reduction
1	Aswan Temple	Entire, circular white to creamy color	Gram positive	Short rods	+	+	+	+	+	−	−
2		Entire, circular white to creamy color	Gram positive	Coccobacilli	−	−	−	+	+	−	−
3		Entire, circular white to creamy color	Gram positive	Rods	+	+	+	+	−	−	+
4		Entire, circular white to creamy color	Gram positive	Short rods	+	+	+	+	−	−	−
5	Edfu Temple	Entire, circular white to creamy color	Gram positive	Coccobacilli	−	+	+	+	−	−	−
6		Entire, circular white to creamy color	Gram positive	Short rods	−	−	−	−	−	−	−
7	Luxor Temple	Entire, circular creamy to yellowish color	Gram positive	Rods	+	+	+	+	−	−	−
8		Entire, circular white to creamy color	Gram positive	Short rods	+	+	+	+	−	−	−
9	Philea Temple	Entire, circular white color	Gram negative	Coccobacilli	−	+	−	−	−	+	+
10		Entire, circular white to creamy color	Gram negative	Coccobacilli	−	+	−	−	−	−	−
11	Komombo	Entire, circular yellow color	Gram negative	Rods	+	+	+	−	−	−	−
12		Entire, circular white to creamy color	Gram negative	Rods	+	−	−	−	−	−	−

### Antimicrobial agents’ producers

#### Antimicrobial activity of the bacterial supernatant

To investigate antimicrobial Activity of the bacterial metabolites, a screening study with the bacterial supernatant (cell-free extract) using the disc diffusion method revealed that isolate number 12 was the most promising producer, which exhibited the largest inhibition zone against *Pseudomonas aeruginosa, Klebsiella Pneumoniae* and *S. aureus* (Table 3). Similarly, the antimicrobial activity of *S. exfoliatus* SAMAH 2021 metabolites were tested against four deteriorative fungal and bacterial strains (*A. niger*, *C. fioriniae*, *P. chrysogenum*, and *L. sphaericus*) which revealed that *P. chrysogenum* was the most susceptible organism followed by *A. niger* and *L. sphaericus* respectively^15^. However, Xi et al.^16^ tested the antimicrobial activities of xenematide B, F and G against six pathogenic bacteria, which revealed that xenematide F showed a potent effect against *P. aeruginosa* with MIC value of 32 μg/mL while xenematide G showed MIC value of 16 μg/mL against *B. subtilis.*

**Table 3:** Screening for the antimicrobial activity of the bacterial metabolites (inhibition zone diameter (mm) ± SD).

Tested pathogen	Isolate number											
	1	2	3	4	5	6	7	8	9	10	11	12
*P. aeruginosa*	6.0 ± 0.2	8.0 ± 0.2	8.0 ± 0.1	7.0 ± 0.3	8.0 ± 0.2	9.0 ± 0.7	9.0 ± 0.1	8.0 ± 0.2	9.0 ± 0.4	10.0 ± 0.1	10.0 ± 0.5	12.0 ± 0.4
*A. baumannii*	6.0 ± 0.4	8.0 ± 0.2	8.0 ± 0.2	8.0 ± 0.2	8.0 ± 0.2	9.0 ± 0.4	9.0 ± 0.4	8.0 ± 0.1	9.0 ± 0.4	8.0 ± 0.3	9.0 ± 0.7	11.0 ± 0.1
*K. pneumoniae*	6.0 ± 0.2	8.0 ± 0.2	8.0 ± 0.2	10.0 ± 0.2	9.0 ± 0.4	9.0 ± 0.4	9.0 ± 0.4	9.0 ± 0.4	9.0 ± 0.4	8.0 ± 0.3	9.0 ± 0.1	12.0 ± 0.3
*P. vulgaris*	7.0 ± 0.3	8.0 ± 0.9	8.0 ± 0.5	8.0 ± 0.4	10.0 ± 0.4	9.0 ± 0.4	9.0 ± 0.4	9.0 ± 0.4	9.0 ± 0.4	10.0 ± 0.4	10.0 ± 0.5	11.0 ± 0.2
MRSA	7.0 ± 0.2	8.0 ± 0.3	9.0 ± 0.7	7.0 ± 0.3	10.0 ± 0.4	9.0 ± 0.4	9.0 ± 0.4	9.0 ± 0.4	9.0 ± 0.4	9.0 ± 0.3	9.0 ± 0.1	11.0 ± 0.4
*E. coli*	6.0 ± 0.4	7.0 ± 0.5	8.0 ± 0.2	9.0 ± 0.4	7.0 ± 0.4	9.0 ± 0.4	9.0 ± 0.4	9.0 ± 0.4	9.0 ± 0.4	8.0 ± 0.6	10.0 ± 0.4	10.0 ± 0.2
*S. aureus*	8.0 ± 0.2	9.0 ± 0.7	10.0 ± 0.1	10.0 ± 0.4	11.0 ± 0.2	9.0 ± 0.4	9.0 ± 0.4	9.0 ± 0.4	9.0 ± 0.4	10.0 ± 0.2	10.0 ± 0.2	13.0 ± 0.30
*C. albicans*	8.0 ± 0.4	8.0 ± 0.2	9.0 ± 0.2	8.0 ± 0.4	7.0 ± 0.3	9.0 ± 0.4	9.0 ± 0.4	9.0 ± 0.4	9.0 ± 0.4	10.0 ± 0.6	10.0 ± 0.4	11.0 ± 0.4

### Identification of the most promising isolate

**Fig. 1:**
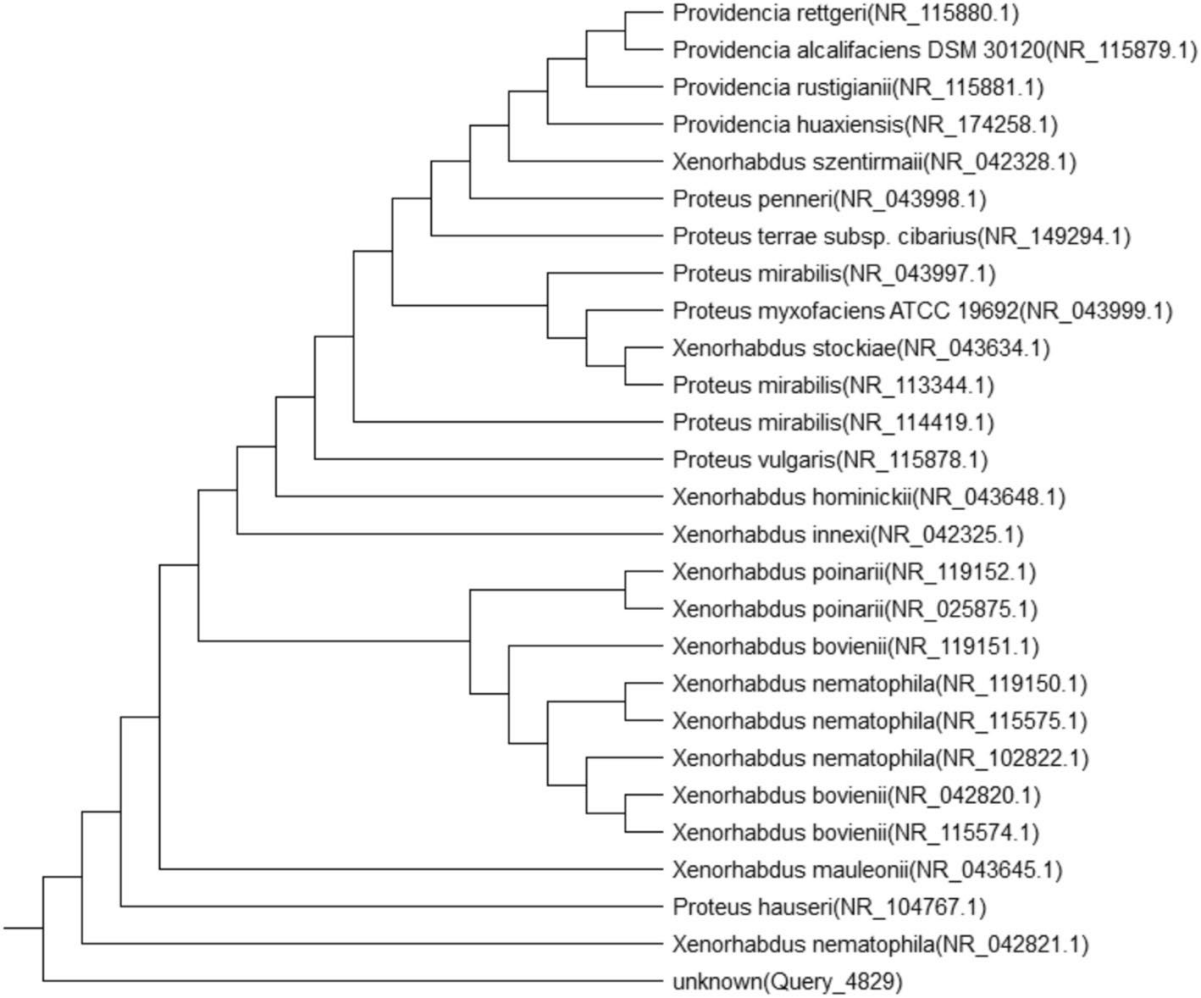
Phylogenetic tree of the most potent strain based on the 16S rRNA gene sequence.

16S rRNA sequencing followed by a multiple sequence alignment was performed in accordance with the National Center for Biotechnology Information (NCBI) database. The promising strain was identified as *Xenorhabdus nematophila* (Fig. 1).

### Optimization of the antimicrobial metabolites activity

#### Optimization using one factor at a time

**Fig. 2:**
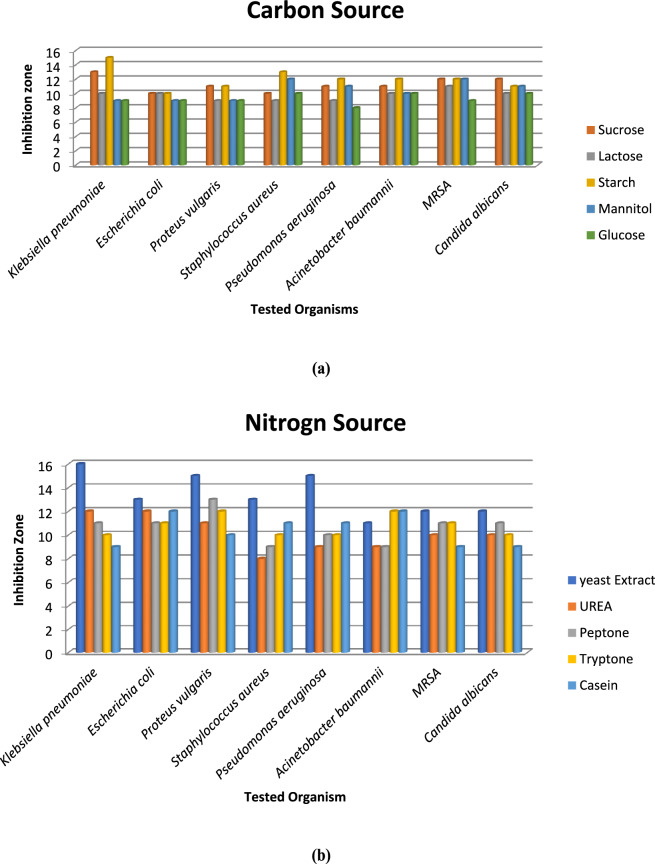
Antibacterial activity of *Xenorhabdus nematophila* as affected by carbon (**a**) and nitrogen (**b**) sources.

Different fermentation parameters, specifically carbon and nitrogen sources, were evaluated to determine the most suitable source that led to the highest antimicrobial activity of the bacterial strain. Results indicated that starch and yeast extract were the optimal carbon and nitrogen sources, respectively, which reported the largest inhibition zone diameters against the tested pathogens (Fig. 2a, b). *E. coli* was one of the most resistant organisms throughout the screening studies, hence, it was chosen for further assessments. Han et al. (2024) discovered that the optimal medium for the production of Xenocoumacin 1 (Xcn 1, antibiotic) from *Xenorhabdus nematophila* YL001 was composed of proteose peptone, maltose, K_2_HPO_4,_ with the addition of arginine to the broth.

#### Central composite design (CCD) optimization

**Fig. 3:**
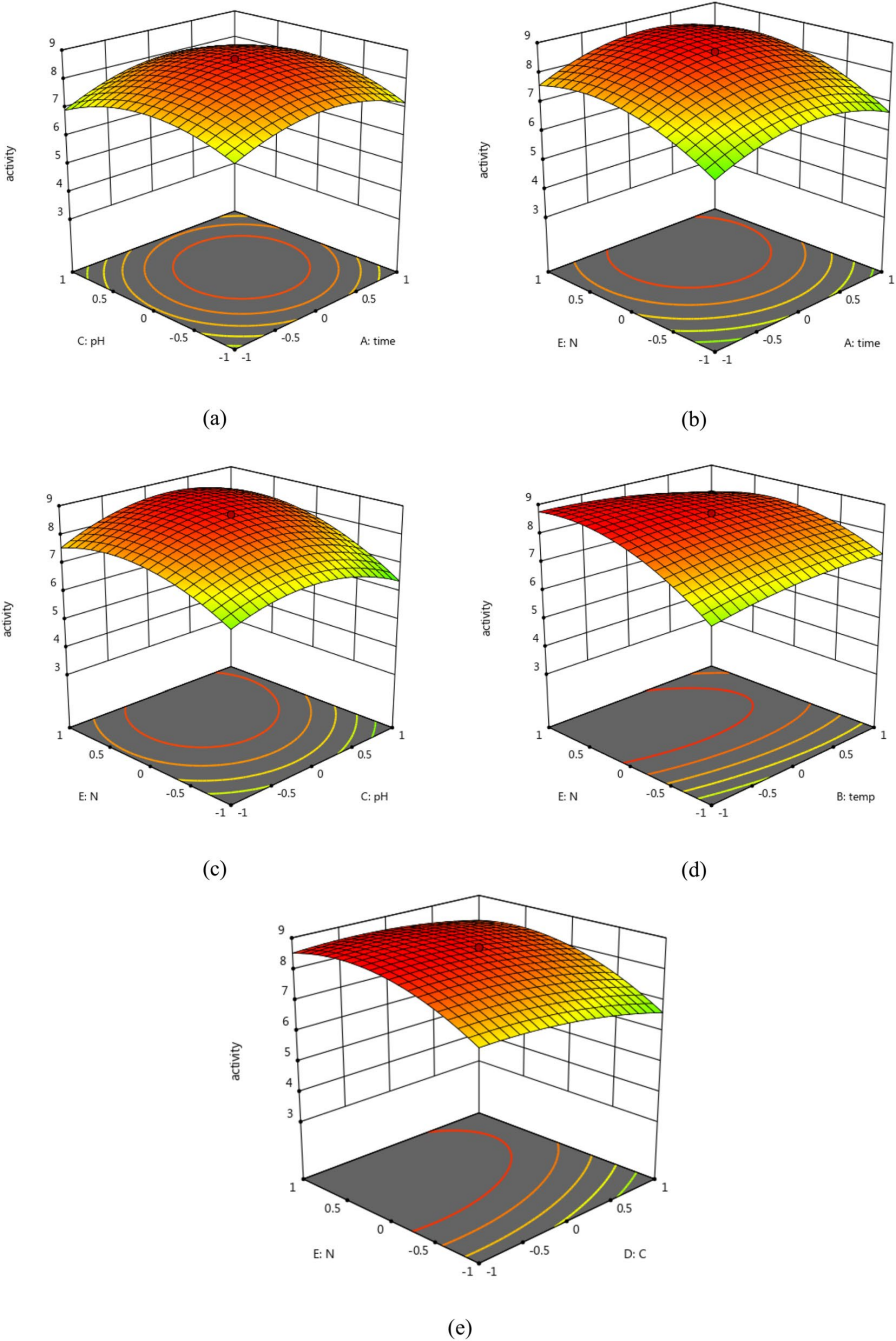
Three-dimensional surface plots for the effects of the tested parameter interactions that lead to the maximum antibacterial effect, where (**a**) shows the interaction between pH and incubation time, (**b**) shows the interaction between nitrogen source concentration and incubation time, (**c**) shows the interaction between nitrogen source concentration and pH, (**d**) shows the interaction between nitrogen source concentration and incubation temperature and (**e**) shows the interaction between nitrogen source concentration, carbon source concentration.

In order to maximize the antimicrobial activity, a central composite design was used. Different fermentation factors were examined at five levels using the Response Surface Methodology (RSM); each variable was studied at five coded levels (− 2.37841, − 1, 0, 1, and 2.37841) for deeper analysis and higher efficiency of the antimicrobial activity. The investigation utilized RSM to examine five factors, with their effects statistically analyzed using Design-Expert 12.0®. Significant factors influencing antimicrobial activity (R1) are shown in Fig. 3, respectively. The significance of each coefficient was determined by Fisher’s F-test and p-values. Results revealed that trial 23 showed the highest antimicrobial activity (Table 4). The optimal conditions for the potent strain were: 0.5% carbon and 0.5% nitrogen sources with an initial pH of 7 for 4 days of incubation at 25 ◦C. ANOVA analysis implied that the linear effect of nitrogen concentration, as well as the significant quadratic effect of time, pH and nitrogen source concentration (p < 0.05) were the significant factors to maximize the antimicrobial activity (R1) (Table 5). Data revealed that pH of the media, incubation time and nitrogen concentrations were significantly affecting the antimicrobial effect of *Xenorhabdus nematophila.*

$$\begin{aligned} Antimicrobial\;Activity = & 8.59172 + 0.186699*A + - 0.0848619*B + 0.0552836*C + - 0.308996*D \\ \quad & + 0.637089*E + 0.25625*AB + 0.1375*AC + 0.04375*AD \\ \quad & + 0.0875*AE + - 0.225*BC + - 0.13125*BD + - 0.3125*BE + - 0.225*CD \\ \quad & + 0.24375*CE + 0.1125*DE + - 0.69846*A^{2} + - 0.203486*B^{2} \\ \quad & + - 0.69846*C^{2} + - 0.230002*D^{2} + - 0.671944*E^{2} \\ \end{aligned}$$

where A is the independent variable of time, B is the temperature, C is the pH, D is the carbon source, and E is the nitrogen source.

**Table 4:** Optimization of the nutritional and environmental factors using central composite design.

Run	A: time	B: temp	C: pH	D: C	E: N	Inhibition zone (mm)
1	1	− 1	1	− 1	− 1	14.8
2	0	0	0	0	0	12.1
3	1	− 1	− 1	1	− 1	14.3
4	− 1	1	1	1	1	14.5
5	1	− 1	1	1	− 1	14.8
6	1	1	1	− 1	− 1	16.5
7	0	0	0	0	0	12.0
8	1	− 1	− 1	− 1	1	17.0
9	1	− 1	1	− 1	1	17.5
10	0	0	0	0	− 2.37841	14.4
11	− 1	1	− 1	1	1	17.1
12	2.37841	0	0	0	0	16.0
13	− 1	1	1	− 1	− 1	14.9
14	0	0	0	− 2.37841	0	17.4
15	0	0	0	0	0	10.7
16	− 1	− 1	1	− 1	1	18.2
17	0	0	0	0	0	11.0
18	− 1	− 1	− 1	1	− 1	15.5
19	1	1	1	− 1	1	17.5
20	− 1	1	− 1	− 1	1	14.8
21	− 1	− 1	− 1	− 1	− 1	14.1
22	0	0	0	0	0	12.1
23	0	0	0	2.37841	0	18.5
24	− 1	1	1	1	− 1	13.5
25	0	0	0	0	2.37841	16.5
26	0	0	0	0	0	12.0
27	− 1	1	− 1	1	− 1	14.9
28	0	− 2.37841	0	0	0	18.2
29	− 1	1	1	− 1	1	17.2
30	1	1	− 1	1	1	14.7
31	1	1	1	1	− 1	14.4
32	1	1	− 1	1	− 1	14.6
33	1	1	1	1	1	17.3
34	0	2.37841	0	0	0	18.0
35	− 1	− 1	1	− 1	− 1	17.6
36	0	0	0	0	0	12.0
37	1	− 1	1	1	1	16.7
38	− 1	− 1	− 1	1	1	15.6
39	0	0	2.37841	0	0	14.5
40	− 1	1	− 1	− 1	− 1	17.1
41	0	0	0	0	0	11.0
42	1	1	− 1	− 1	1	16.1
43	− 1	− 1	1	1	− 1	14.3
44	1	− 1	− 1	1	1	16.4
45	1	− 1	− 1	− 1	− 1	14.1
46	− 1	− 1	1	1	1	17.1
47	− 2.37841	0	0	0	0	13.6
48	0	0	− 2.37841	0	0	16.1
49	− 1	− 1	− 1	− 1	1	17.3
50	1	1	− 1	− 1	− 1	17.0

**Table 5:** ANOVA analysis.

Source	Sum of squares	df	Mean square	F-value	*p*-value	
Model	98.71	20	4.94	3.80	0.0006	Significant
A-time	1.51	1	1.51	1.16	0.2896	
B-temp	0.3119	1	0.3119	0.2404	0.6276	
C-pH	0.1324	1	0.1324	0.1020	0.7517	
D-C	4.14	1	4.14	3.19	0.0847	
E-N	17.58	1	17.58	13.55	0.0009	
AB	2.10	1	2.10	1.62	0.2133	
AC	0.6050	1	0.6050	0.4663	0.5001	
AD	0.0612	1	0.0612	0.0472	0.8295	
AE	0.2450	1	0.2450	0.1888	0.6671	
BC	1.62	1	1.62	1.25	0.2730	
BD	0.5512	1	0.5512	0.4249	0.5196	
BE	3.13	1	3.13	2.41	0.1315	
CD	1.62	1	1.62	1.25	0.2730	
CE	1.90	1	1.90	1.47	0.2358	
DE	0.4050	1	0.4050	0.3122	0.5806	
A^2^	27.11	1	27.11	20.90	< 0.0001	
B^2^	2.30	1	2.30	1.77	0.1933	
C^2^	27.11	1	27.11	20.90	< 0.0001	
D^2^	2.94	1	2.94	2.27	0.1431	
E^2^	25.09	1	25.09	19.34	0.0001	
Residual	37.62	29	1.30			
Lack of fit	37.62	22	1.71			
Pure error	0.0000	7	0.0000			
Cor total	136.33	49				

Surface plots represented in Fig. 3 showed that the color gradient (from red to green) indicates the levels of activity, with higher values around the center and lower values towards the edges. Moreover, the horizontal plane in the contour plots at the bottom shows contour lines, which represent lines of constant activity. Each contour line helps visualize the activity level without the 3D perspective, making it easier to see how changes in the nitrogen source concentration, incubation time and pH affect the antibacterial activity. The peak of the surface plot indicates the optimal combination of the two tested variables that yields the maximum activity. Hence, we can determine the best settings for the inputs (significant factors) to achieve the highest possible outcome (antibacterial activity). Sa-uth et al.^17^ reported that the modifying growth parameters such as pH, incubation temperature, agitation, aeration, and nutrients greatly affect the production of secondary metabolites by *Xenorhabdus* spp. The enhancement of antibiotic production by *X. nematophila* can be achieved by controlling the pH of the growth medium^18,19^. Altering the pH level from 6.5 to 7.5 during mid-exponential significantly increased antibiotic synthesis up to 185%. Wang et al.^20,21^ observed that when the bacterial strains of *X. nematophila* were incubated at 28.5 °C with increased levels of dissolved oxygen the antibiotic biosynthesis was improved. Du et al.^22^ documented an increase in antibiotic synthesis when *X. bovienii* was cultivated in high NaCl concentrations. Comparable findings were reported by Crawford et al.^23^. The addition of sorbitol to the growth medium led to a reduction in antibiotic synthesis^23^. Both carbon and nitrogen levels are equally significant. According to Wang et al.^18,19^ *X. bovienii* exhibited the greatest amounts of antimicrobial agents’ production when cultured in the presence of glycerol and soytone. The production of antibiotic by *X. nematophila* was enhanced when cultivated in the presence of glucose and peptone^20,21^. According to Sa-uth et al.^17^, the cultivation of *X. stockiae* in the presence of sucrose and yeast extract resulted in an augmentation in antibiotic productivity. Thus, it is evident that these organisms are selective regarding the origin and the carbon and nitrogen ratio.

### Antimicrobial metabolites identification and characterization

**Fig. 4:**
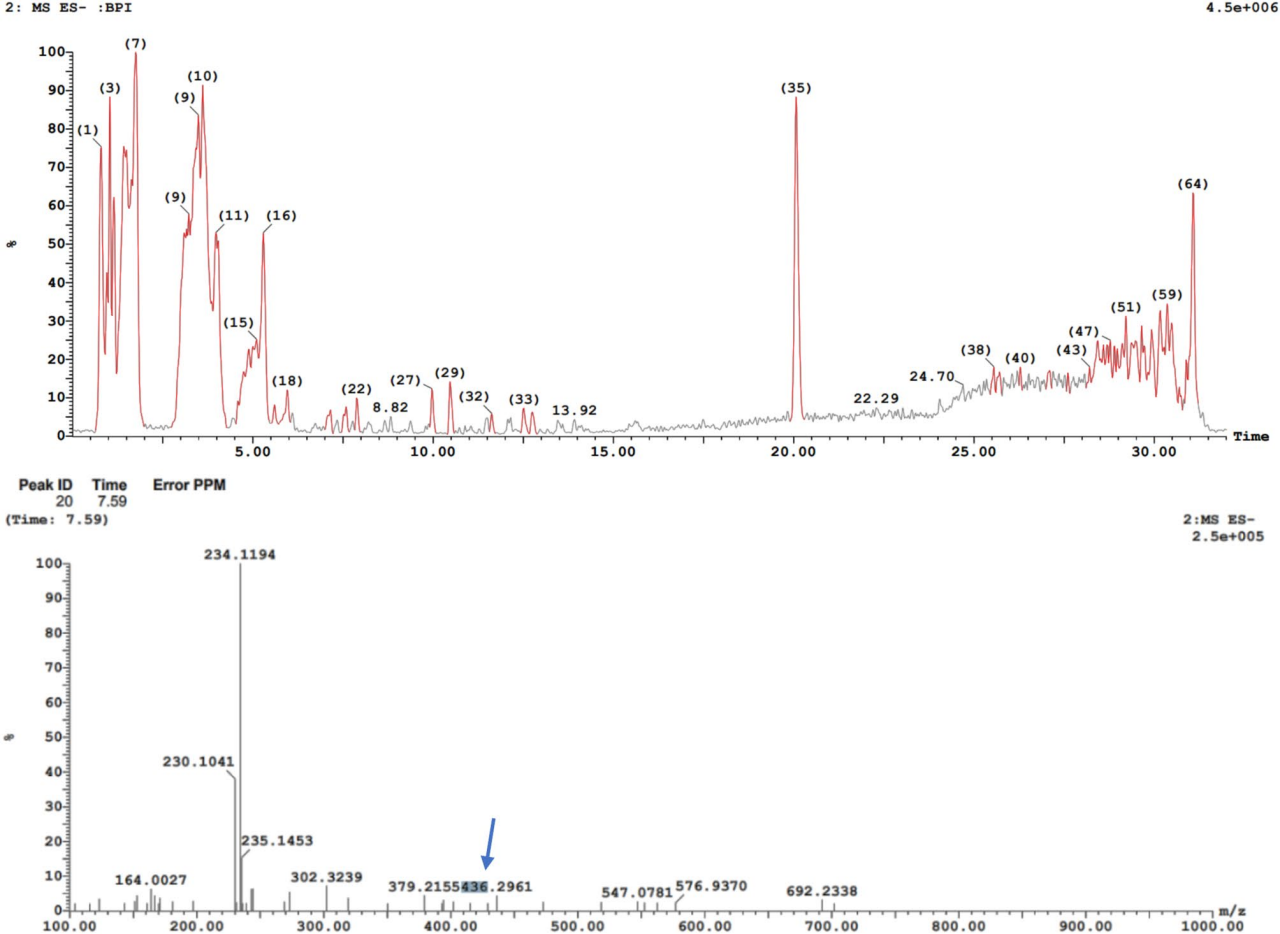
Bioactive compounds of *Xenorhabdus nematophila*: LC/MS (**a**), MS/MS spectra of Xenortide at 7.59 min (**b**).

**Fig. 5:**
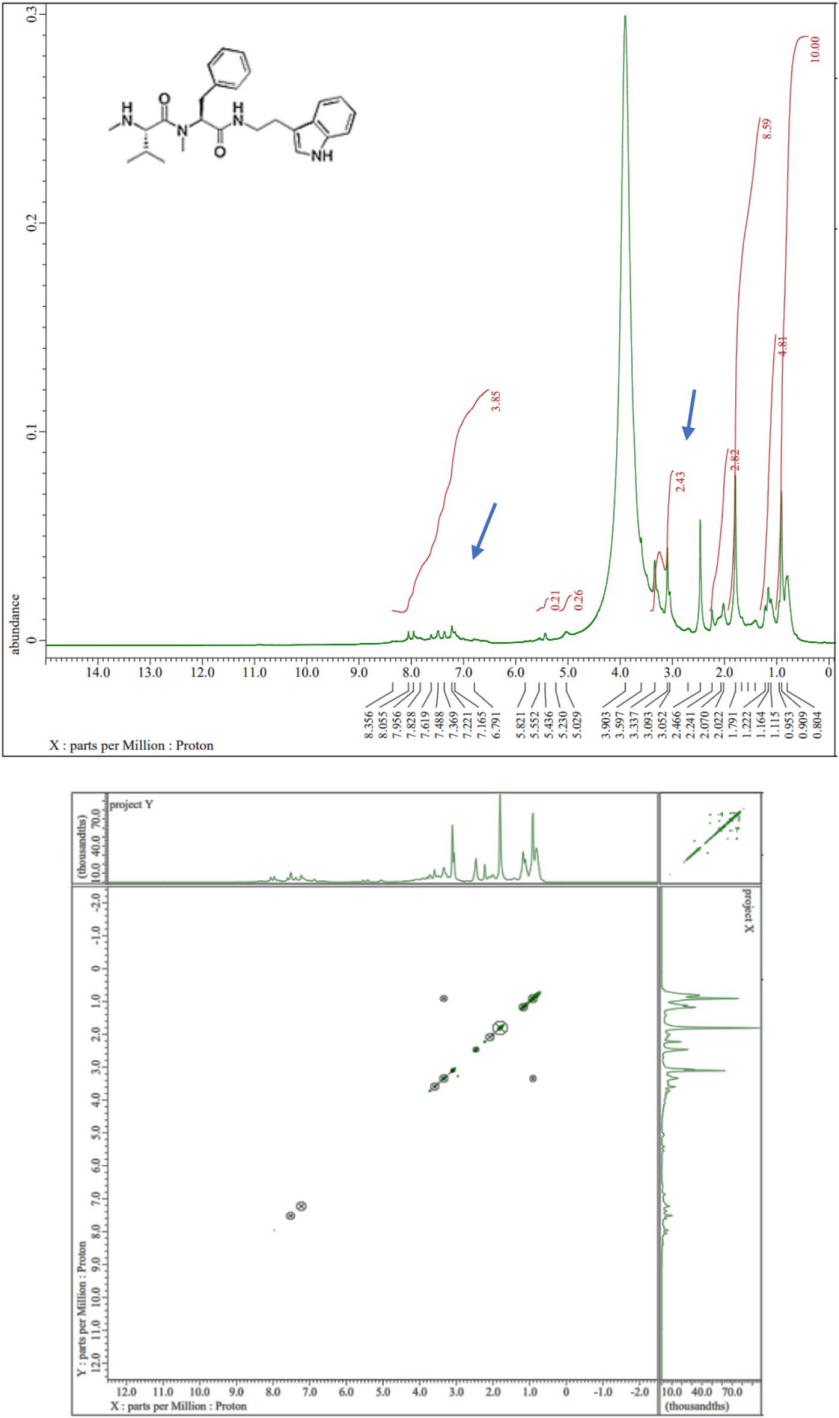
NMR study of *Xenorhabdus nematophila* bioactive compound.

LC/MS analysis revealed that the *Xenorhabdus nematophila* bioactive metabolites was identified as Xenortide (Fig. 4 a, b) when compared with the MassBank database. The most abundant ions [M + H] + were 436.29 m/z. Reimer et al.^24^. The detection of a new derivative named xenortide D (4, C26H35N4O2, m/z 435.2755 [M + H] +), in addition to the known xenortides A − C (1 − 3), although this derivative is produced in only trace amounts. Briefly, a comparison of the MS2 fragmentation pattern of xenortides A − C with D indicated that aline and phenylalanine served as building blocks of xenortide D, which was confirmed by labelling with L-[2,3,4,4,4,5,5,5-2H8]valine and L-[2,3,3,5,6,7,8,9-2H8]-phenylalanine fed to a culture of *X. nematophila* HGB081 in LB medium. Analysis of the COSY data (Fig. 5) attributed the phenyl groups to one phenylalanine and one phenethylamine residue, in accordance with Lang et al.^25^. A set of peaks at 6.8–7.5 ppm (Aromatic Region) suggests the presence of an aromatic ring, with multiple protons indicated, with one substituent being an alcohol and another being an alkyl chain. Peaks in the range of 2.0–3.0 ppm might indicate protons attached to a carbon chain or possibly to a carbon adjacent to a functional group. A cluster of peaks was noticed at 0.5–1.5 ppm, suggesting several protons from the aliphatic chain, integrating to a total of 10 protons. The integration values align with the proposed number of protons: Aromatic protons (4), Aliphatic/Alkanol protons (3), and Aliphatic chain protons (10). The COSY spectrum displays cross-peaks between aromatic protons and additional protons on a carbon chain, confirming direct connectivity. Correlations between protons in the 2.0–3.0 ppm range indicate proximity to both aromatic and aliphatic groups, suggesting these protons might belong to a carbon adjacent to both the aromatic ring and a functional group. Based on the combined analysis of the NMR and COSY spectra, the proposed structure of xenortide D could be summarized as follows: an alcohol group at a position adjacent to the aromatic ring and a branched or straight-chain aliphatic group whose protons are well represented in both NMR and COSY correlations. The structure showcases a classic aromatic framework with distinct functionalization, suggesting potential biological activity related to the modifications present.

### Nano-xenortide synthesis and characterization

**Fig. 6:**
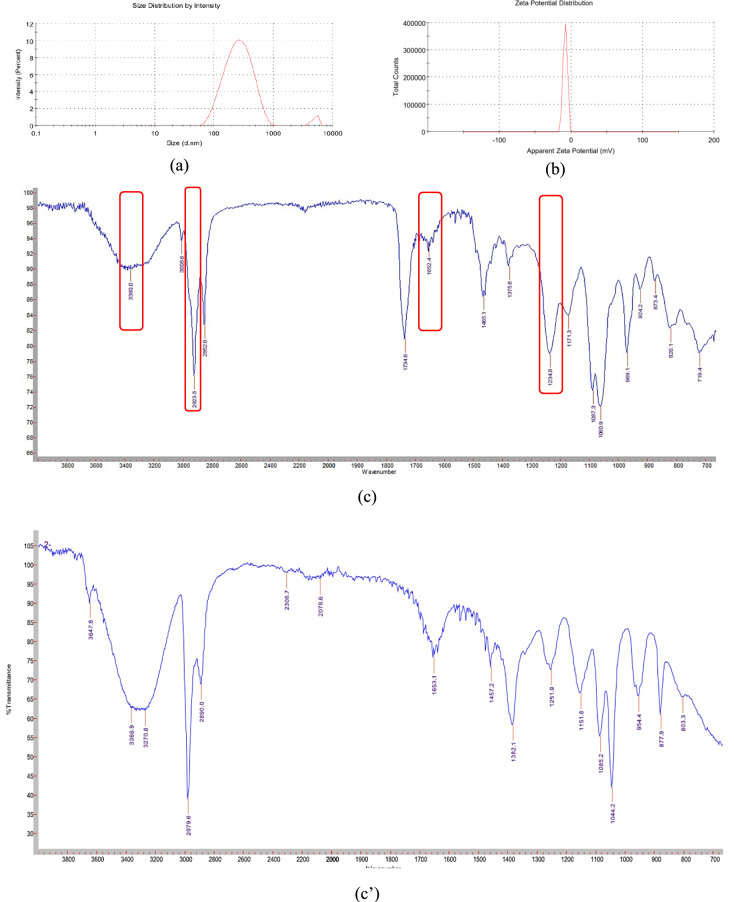

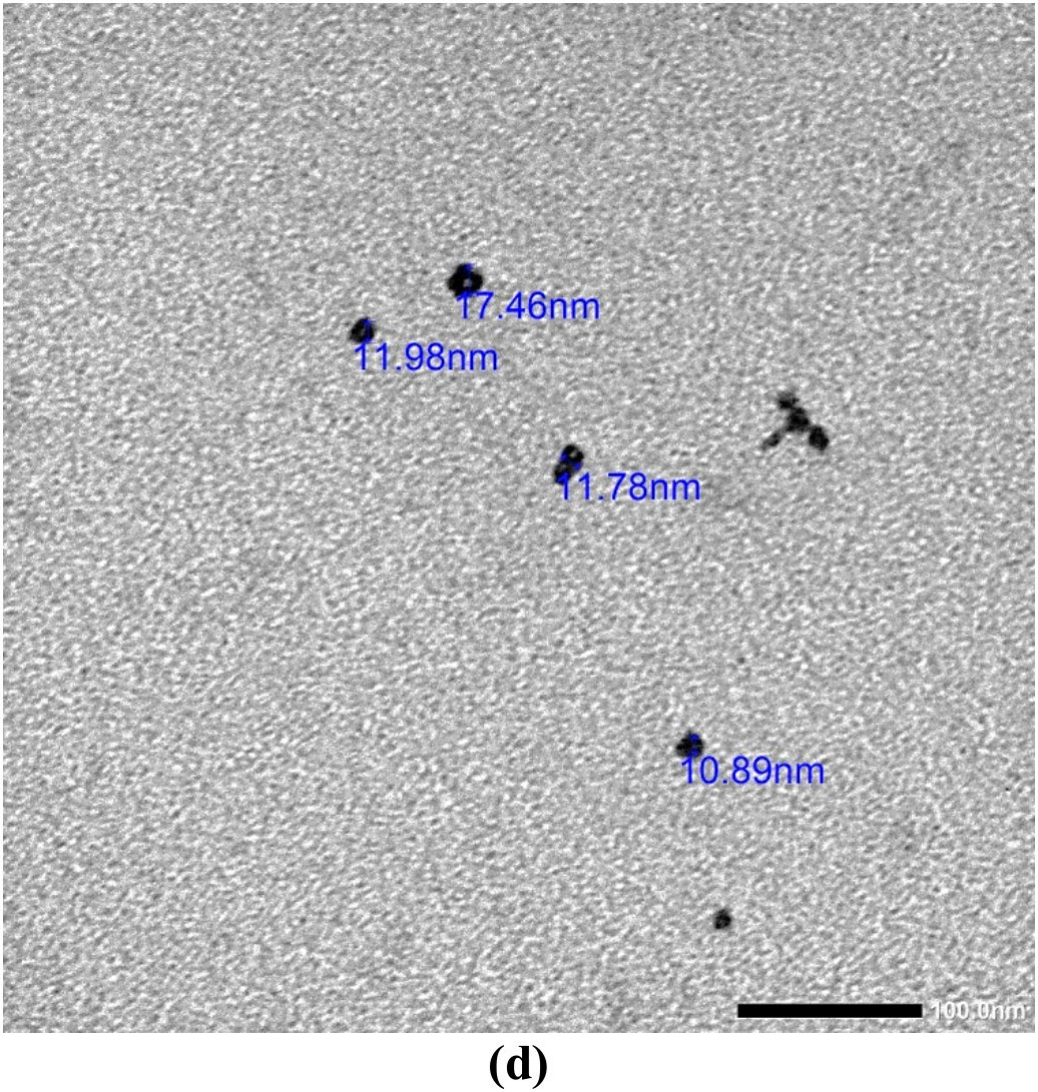
Physicochemical properties of the nano-xenortide zeta size (**a**) and potential (**b**), FTIR spectra of xenortide (**c**) and Nano-xenortide (**c**′), and TEM micrograph (**d**).

The nanosynthesis was conducted using the ball-milling technique, and the resulting nanoparticles were characterized through zeta potential, PDI, FTIR, and a transmission electron microscope (TEM) to determine their nanoparticles’ shape, size, homogeneity, and stability (Fig. 6). C=O stretching from the carbonyl group of the amide bond was noticed at 1652.4 cm^-1^. After ball milling (nano-Xenortide formation), increased peak intensity was noticed, possibly indicating better exposure or dispersion of certain functional groups. While shifts in the wavenumbers of certain peaks (e.g., amide I, amide III) towards higher values indicate the formation of new interactions (Table 6). The position of the C-H stretching peaks might also reflect changes in the methyl or methylene groups due to the mechanical processing. It was found that the newly synthesized nano-xenortide had a circular shape with an average size of ± 13 nm (as detected by the TEM investigation). The zeta potential and PDI were − 8.5 mV, and 2.93, respectively, indicating the relative stability of nano-xenortide.

**Table 6:** FTIR spectra before and after ball milling of xenortide.

Observed peaks	Xenortide (before milling (cm^−1^))	Nano-xenortide (after milling (cm^−1^))	Observation
Amide I (C=O Stretch)	1652.4	1653.1	Minimal shift; peak remained strong
Amide III	1234.8	1251.9	Shift indicates structural changes
N–H stretch	3360.0	3370.8	Broader peak, possibly indicating new hydrogen bonding
C–H stretch	2923.5	2979.6	Possible shift due to improved dispersion

### Antimicrobial activity of the synthesized nano-xenortide

**Fig. 7:**
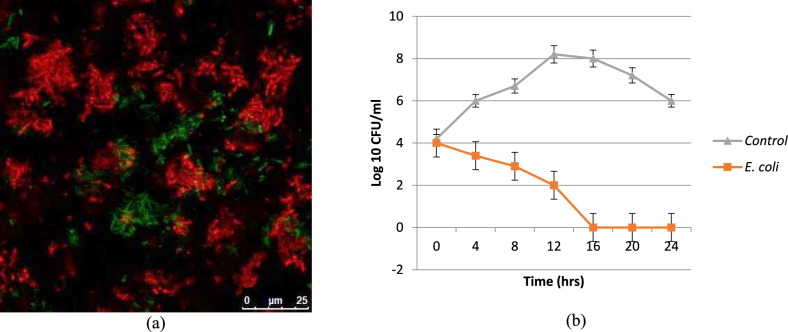
Antibacterial effect of the synthesized Nano-xenortide: confocal microscope study (**a**) and time-kill curve (**b**) of the treated cells.

The antimicrobial activity of the synthesized nano-Xenortide was evaluated using disc diffusion, MIC, time–kill curve and confocal microscopy. The results revealed that the synthesized nano-Xenortide showed significant antibacterial activity with inhibition zone diameters of 19 mm against *E. coli*. On the other hand, the MIC value was 20 µg/ml. Combinations of nano-Xenortide with Ampicillin and Gentamicin exhibited a synergistic effect. (Table 7). Confocal microscope assessment showed that most of the cells were dead (red color) and only a few cells were still alive after the incubation period (green color) (Fig. 7a). It was shown that *E. coli* was completely eradicated after 16 h incubation with the prepared nanoparticles (Fig. 7b).

**Table 7:** Antibacterial effect of nano-xenortide combined with commonly known antibiotics.

Tested antibacterial agent	Inhibition zone diameter (mm)	Inhibition zone diameter (mm) of the combination	Combination effect
Nano-xenortide	19.0	–	–
Ampicillin	12.0	32.0	Synergy
Trimethoprim-sulfamethoxaxole	22.0	22.0	Antagony
Gentamicin	11.0	33.0	Synergy
Amoxicillin-clavulanic acid	30.0	30.0	Antagony
Oxacillin	20.0	20.0	Antagony
Polymxyin B	16.0	18.0	Antagony

### Cytotoxicity

In a trial to assess the cytotoxic potency of Nano-xenortide, different cell lines of human tumours and normal cells were investigated. The inhibition of cell proliferation and induction of cell death were observed. The data revealed that the cytotoxic effects of Nano-xenortide on normal cells and cancer cells were directly proportional to the nanoparticles concentration. The IC50 of Nano-xenortide against normal cells, namely W138 and Vero models, were 4027 and 3910 µg/mL, respectively. On the other hand, the efficacy of the synthesized Nano-xenortide against cancer cells, namely Caco-2 demonstrated significant potential for use as a therapy against colorectal carcinoma (Table 8). The calculated selectivity index indicates that the Nano-xenortide was safe and effective. Esmati et al.^26^ reported that the structure–activity relationship (SAR) study demonstrated that tryptamides Xenortide (Xen) B and D were more active than phenylethylamides Xenortide A and C. Furthermore, ( −)-Xen B (IC50 = 19–25 μM) and ent-( +)-Xen D (IC50 = 21–26 μM) exhibited the highest cytotoxicity while remaining non-toxic to normal cells. Notably, the SAR results provided by Esmati et al.^26^ indicated that the stereochemistry at C8 and C11 in ( −)-Xen B and ent-( +)-Xen D play a critical role in cytotoxic activity.

**Table 8:** IC50 and the selectivity index of Nano-xenortide against Caco-2 72 h.

Sample	IC 50 (µg/mL)			Caco-2 selectivity index with	
	WI38	Vero	Caco-2	Vero	WI38
Nano-xenortide	4027 ± 69.32	3910 ± 98.21	573 ± 43.98	7.02 ± 0.52	6.82 ± 0.32

Data are represented as mean ± SD, n = 3.

## Conclusion

Data of the present investigation concluded that:

- *Xenorhabdus nematophila* was isolated from Egyptian ancient site soil samples and was selected as a promising antibacterial agent producer
- Xenortide was detected as a bioactive molecule in the bacterial secondary metabolites in the culture filtrate.
- Central composite statistical design for the optimization of the bioactive agent production revealed that the best fermentation parameters were carbon and nitrogen concentration and pH.
- Nano-xenortide produced by ball milling was more effective against the tested pathogens.
- Cytotoxicity test showed that nano-xenortide might be a safe antibacterial and anti-colorectal-carcinoma agent.

## Data Availability

All the original data are available upon reasonable request for correspondence authors.
